# The extent of intestinal involvement is closely related to the severity of IgAV: a risk stratification study based on CT

**DOI:** 10.1080/07853890.2025.2462260

**Published:** 2025-02-07

**Authors:** Yuanqiu Li, Ziman Xiong, Yufan Wang, Yuchen Jiang, Yaqi Shen, Xuemei Hu, Daoyu Hu, Zhen Li

**Affiliations:** ^a^ Department of Radiology, Tongji Hospital, Tongji Medical College, Huazhong University of Science and Technology, Wuhan, China; ^b^ Department of Radiology, National Medical Center for major public health events, Tongji Hospital, Tongji Medical College, Huazhong University of Science and Technology, Wuhan, China

**Keywords:** Immunoglobulin A vasculitis, computed tomography, risk stratification, quantitative, D-dimer

## Abstract

**Objectives:**

To analyze the differences in clinical manifestations between children and adults with intestinal involvement in IgAV and to identify the specific subtypes requiring particular attention.

**Methods:**

A systematic review of the HIS system was conducted on patient data from four centers at Tongji Hospital between January 2015 and October 2022. Patients with a diagnosis of IgAV with intestinal involvement were further analyzed. Clinical manifestations, laboratory data, and CT findings at the time of initial hospitalization were recorded. The differences in clinical manifestations between children and adults were analyzed. The extent of intestinal involvement, linked to disease severity, was quantitatively assessed by evaluating the number of affected intestinal segments on CT imaging. Laboratory markers that could reflect severe intestinal involvement were explored. Furthermore, patients were classified based on the sites of involved intestinal segments: L1 type (duodenum and/or jejunum), L2 type (ileum), L3 type (duodenum and/or jejunum and ileum), and L4 type (limited to the colorectum). A comparison of the first three types was performed.

**Results:**

A total of 148 patients were enrolled (67 children and 81 adults). The proportion of joint pain and renal involvement was higher in adults. D-dimer level was an independent risk factor for severe intestinal involvement (OR = 1.104, *p* = .016). In the first three types patients based on the sites of involved intestinal segments, it found that L3 type patients had a longer hospital stay.

**Conclusion:**

With the exception of joint pain and renal involvement, there were no significant differences in clinical symptoms between children and adults. CT imaging provided objective insights into the extent of intestinal involvement, which correlated with disease severity. Patients with widespread small bowel involvement displayed a more severe disease state.

## Introduction

Immunoglobulin A (IgA) vasculitis (IgAV), also known as Henoch-Schönlein purpura (HSP), is the most common systemic vasculitis in children and can also affect adults. The exact pathogenesis of IgAV was unknown. But it was believed to be associated with environmental factors, genetic factors, innate and acquired immune abnormalities, and the role of IgA1 immune complexes in individuals with lactose intolerance [1,2]. IgAV primarily affected children aged 3–15 years, with an incidence rate of approximately 15/100,000–20/100,000. The incidence rate was even lower in adults, at approximately 10/100,000 [3]. In addition to differences in the incidence rate, the severity of clinical manifestations also varied between adults and children [4].

Approximately 51%–56% of IgAV patients had gastrointestinal involvement [5]. The short-term prognosis of patients depended on the severity of acute gastrointestinal involvement. At present, the diagnostic criteria for IgAV are mostly based on clinical manifestations and pathological findings [6–8]. However, the clinical manifestations in children are non-specific and may simply present as crying or fussiness. This makes it challenging for clinicians to promptly diagnose the condition, assess its severity, and administer timely and appropriate treatment. Previous definitions of severe intestinal involvement in patients were mainly based on clinical symptoms or the presence of intestinal edema on imaging [9–11]. However, due to the subjectivity of clinical symptoms and the lack of rigor in medical record documentation, more objective indicators were needed to assess the severity of intestinal involvement. The acquisition of quantitative indicators from imaging examinations, such as using the extent of intestinal involvement to reflect the severity of intestinal involvement, provided a potential solution. Ultrasound and computed tomography (CT) are commonly used for IgAV patients suspected of intestinal involvement [12,13]. While ultrasound avoids radiation exposure, it has several limitations, including lower image resolution, sensitivity to interference from intestinal gas, and significant operator dependency. In contrast, CT overcomes these limitations and provides a reliable tool for risk stratification in patients with intestinal involvement. Additionally, the development of economical and practical biomarkers that accurately predict and rapidly diagnose severe intestinal involvement in IgAV patients is also crucial. A meta-analysis showed that a higher neutrophil-to-lymphocyte ratio (NLR) and lower mean platelet volume (MPV) indicated more severe intestinal involvement in children [14]. IgAV patients may experience small vessel spasm, platelet aggregation, and thrombus formation. Therefore, D-dimer was commonly used as a molecular marker for the diagnosis of IgAV.

Among IgAV patients, the small intestine was the most commonly involved segment due to its susceptibility to ischemic injury [15]. The duodenum and jejunum were the two most frequently affected segments. Analyzing the clinical manifestations and disease severity of patients can provide valuable insights. Specifically, focusing on the involved segments of the intestinal tract can help stratify patients for better management. Additionally, this analysis can contribute to optimizing therapeutic outcomes.

Therefore, this study aimed to summarize the imaging features in IgAV patients with intestinal involvement and analyze the differences in clinical manifestations between children and adults at four centers in Tongji Hospital. It aimed to determine the severity of intestinal involvement in IgAV patients based on quantitative imaging indicators, explore laboratory indicators that can reflect the severity of intestinal involvement, and investigate specific types of intestinal involvement that require more clinical attention.

## Materials and methods

### Patient information

A retrospective review was conducted on patients diagnosed with intestinal involvement in IgAV at Tongji Hospital from January 2013 to October 2022. The Ethics Committee of Tongji Hospital, Tongji Medical College, Huazhong University of Science and Technology approved this study and waived the informed consent (TJ-IRB202404058). Patient privacy was strictly protected, and no identifying information was disclosed. This study followed the principles of the Declaration of Helsinki. The diagnosis of IgAV was based on the criteria established by the American College of Rheumatology [6,7] or the European League Against Rheumatism and the Pediatric Rheumatology European Society [8], or it can be diagnosed through discussion by a multidisciplinary team. Patients diagnosed with other diseases were excluded. Intestinal involvement was defined by abdominal pain, nausea or vomiting, diarrhea, bowel obstruction, intussusception, gastrointestinal bleeding, intestinal ulcers, intestinal perforation, and intestinal necrosis [16]. Exclusion criteria were as follows: (1) unavailable CT scans and (2) patients with concomitant immune disorders.

The medical records of initial hospitalizations were evaluated. The data including age, gender, length of hospital stay, prodromal symptoms (upper respiratory tract infection), skin purpura, joint pain, renal involvement (defined as hematuria and/or proteinuria [8,17]), fever, weight loss, abdominal pain, diarrhea, nausea and vomiting, gastrointestinal bleeding (melena or positive fecal occult blood), and whether abdominal symptoms were the initial presentation were recorded. Laboratory test results from the initial examination were also documented, including D-dimer, C-reactive protein (CRP), erythrocyte sedimentation rate (ESR), albumin (Alb), hemoglobin (Hb), white blood cell (WBC), neutrophils, lymphocytes, and MPV. Children were patients with age <18 years. Adults were patients with age ≥18 years.

### Imaging data

**Figure 1.**
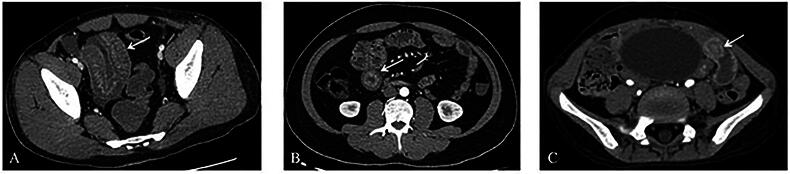
The imaging features of IgAV patients. (A) petaloid bowel wall thickening; (B) target sign; (C) ileo-ileal intussusception. IgAV: immunoglobulin a vasculitis.

CT scans, including non-enhanced or enhanced scans, were performed during the initial hospitalization. Enhanced scans were preferred for better visualization of the affected intestinal segments. If a patient had both non-enhanced and enhanced CT scans during the initial hospitalization, the enhanced scan was selected for image analysis. The intestine was divided into the duodenum, jejunum, proximal ileum, ileocecal region (terminal ileum and cecum), ascending colon, transverse colon, descending colon, sigmoid colon, and rectum. Two experienced abdominal radiologists independently assessed the affected intestinal segments and determined whether patients presented with petaloid bowel wall thickening, target sign (Figure 1). And any disagreements were resolved through consultation. The occurrence of intestinal complications, such as intussusception, intestinal necrosis, and intestinal perforation, was also evaluated.

### Grading of intestinal involvement severity

The extent of intestinal involvement was linked to intestinal involvement severity. We scored each affected intestinal segment mentioned above. Since the jejunum was the most involved segment, it was scored as 1 if the involved length was <20 cm and 2 if it was ≥20 cm. The remaining intestinal segments were each scored as 1. Patients with a total score <3 were classified as mild intestinal involvement, while those with a score ≥3 were classified as severe intestinal involvement. Then, we explored the laboratory indicators associated with severe intestinal involvement.

### Classifications based on the sites of intestinal involvement

**Figure 2.**
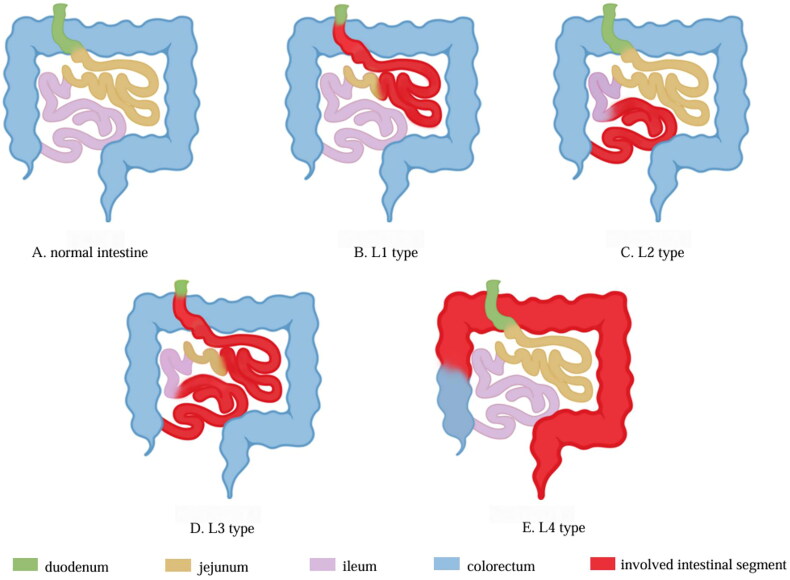
Schematic diagram of IgAV intestinal involvement classification. L1 type: involvement of the duodenum and/or jejunum; L2 type: involvement of the ileum; L3 type: involvement of the duodenum and/or jejunum and ileum; L4 type: isolated involvement of the colorectum. IgAV: immunoglobulin a vasculitis.

With reference to the Montreal classification of Crohn’s disease, we classified IgAV patients according to the sites of intestinal involvement (Figure 2): L1 type: involvement of the duodenum and/or jejunum; L2 type: involvement of the ileum; L3 type: cc; L4 type: isolated involvement of the colorectum. Clinical characteristics and length of hospital stay differences among different types of patients were investigated.

### Statistical analysis

Statistical analysis was performed using SPSS (version 25.0). Continuous variables were presented as mean ± standard deviation if they followed a normal distribution. Otherwise, they were presented as median (interquartile range). Categorical variables were presented as n (%). Independent sample t-tests or Mann-Whitney U tests were used for continuous variables. While χ^2^ tests or Fisher’s exact tests were used for categorical variables. Kappa statistics were used to assess the agreement between two radiologists. The degree of agreement was determined as follows: ≤0.20, slight agreement; 0.21–0.40, fair agreement; 0.41–0.60, moderate agreement; 0.61–0.80, good agreement; and >0.80, excellent agreement.

For the laboratory parameters with missing values, multiple imputation was performed using the multiple imputation method. The imputation process was repeated 5 times, and the imputed datasets were combined to obtain complete data. Univariate logistic regression analysis was performed on the laboratory parameters. Variables with a *p* value <.1 were included in the multivariate logistic regression analysis to identify independent risk factors for severe intestinal involvement.

For the analysis of the L1–L4 types of patients and related factors, the Kruskal-Wallis H test was used for continuous variables in the multiple groups’ comparison. The *χ*^2^ test was used for categorical variables. If the *p*-value was < .05, Bonferroni correction was applied for pairwise comparisons.

## Results

**Figure 3.**
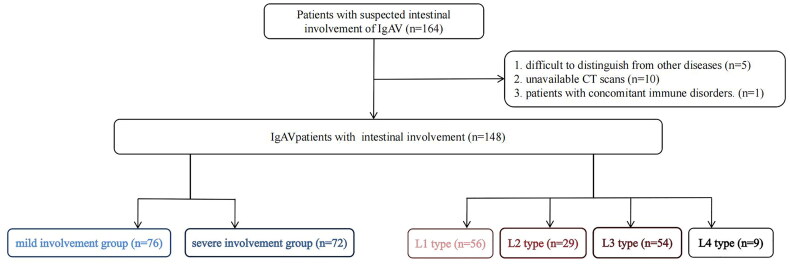
The flowchart of the study. L1 type: involvement of the duodenum and/or jejunum; L2 type: involvement of the ileum; L3type: involvement of the duodenum and/or jejunum and ileum; L4 type: involvement of the colorectum. IgAV: immunoglobulin a vasculitis; c.

**Figure 4.**
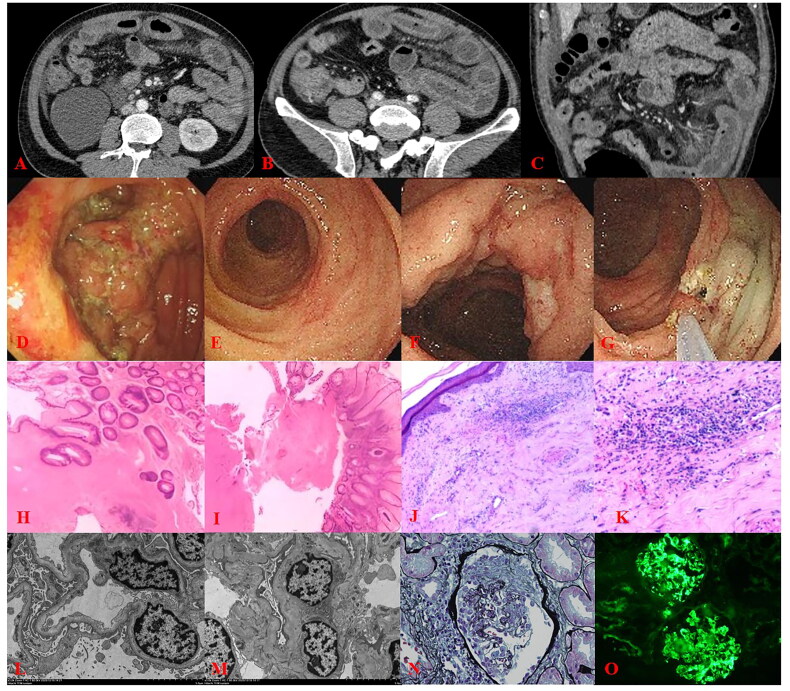
The patient presented with mucopurulent bloody stools for 5 days without apparent cause before seeking medical attention. He experienced systemic joint pain, followed by hematuria, proteinuria, and the development of purpura rash on his limbs. Skin biopsy showed findings consistent with leukocytoclastic vasculitis. (A–C) Enhanced CT scan revealed thickening and enhancement of the intestinal walls in the jejunum, ileum, and cecum. (D) Colonoscopy reached the cecum and indicated a neoplasm near the appendiceal orifice with visible ulceration on the surface. Colonoscopy was repeated 18 days later, and the scope reached the terminal ileum. (E) Scattered ulcers were observed in the terminal ileum, with one displaying a longitudinal pattern, some in the healing phase. (F and G) The ascending Colon exhibited irregular mucosal hyperplasia and edema, along with scattered irregular and larger circular ulcers. (H) Pathology showed chronic active enteritis with ulceration in the terminal ileum (40× magnification). (I) Pathology showed chronic enteritis with ulceration and occlusion of small vessels in the rectum(40× magnification). (J and K) skin biopsy showed findings consistent with leukocytoclastic vasculitis (40× magnification and 100× magnification, respectively). Renal biopsy findings: (L and M) electron microscopy suggested fusion of foot processes and the presence of electron-dense deposits (3000× magnification). (N) Light microscopy after Masson’s staining revealed crescent formation in the renal glomeruli (400× magnification). (O) Immunofluorescence displayed granular deposits in the mesangial area, with IgA+++ staining (200× magnification). CT: computed tomography.

A total of 148 patients were enrolled, including 67 children and 81 adults. The flowchart was shown in Figure 3. An IgAV patient with enhanced CT images, endoscopy and pathological results was shown in Figure 4.

### Clinical data

The clinical data for patients were shown in Table 1. The median age of the children was 12.00 years, while the median age of the adults was 34.00 years. The proportions of joint pain and renal involvement were higher in the adults (13.4% vs. 34.6%, *p* = .003; 34.3% vs. 54.3%, *p* = .015). There were no significant differences in other manifestations.

**Table 1.** Clinical date of IgAV patients.

Item	Children (*n* = 67)	Adults (*n* = 81)	*p*
Age (year)	12.00 (6.00, 15.00)	34.00 (24.00, 53.50)	
Gender (male)	45 (67.2%)	59 (72.8%)	.452
Length of hospital stay (day)	13.00 (9.00, 21.00)	12.00 (8.00, 18.50)	.468
Prodromal symptoms (upper respiratory tract infection)	3 (4.5%)	11 (13.6%)	.060
Skin purpura	56 (83.6%)	69 (85.2%)	.789
Joint pain	9 (13.4%)	28 (34.6%)	.003
Renal involvement	23 (34.3%)	44 (54.3%)	0.015
Fever	13 (19.4%)	12 (14.8%)	.458
Weight loss	8 (11.9%)	13 (16.0%)	.476
Abdominal pain	63 (94.0%)	80 (98.8%)	.176
Diarrhea	48 (71.6%)	64 (79.0%)	.298
Nausea and vomiting	32 (47.8%)	38 (46.9%)	0.918
Gastrointestinal bleeding	54 (80.6%)	62 (76.5%)	.551
Melena	26 (48.1%)	45 (72.6%)	
Positive fecal occult blood	28 (51.9%)	17 (27.4%)	
Abdominal symptoms as the initial presentation	27 (40.1%)	26 (32.1%)	.300

IgAV: immunoglobulin A vasculitis.

### Imaging features

There were 126 enhanced CT scans and 22 non-enhanced CT scans included for analysis. In the total 148 patients, petaloid bowel wall thickening was observed in the intestines of 58 patients (Kappa value = 0.900). In 126 patients with enhanced CT scans, 83 patients presented with target sign (Kappa value = 0.930).

### Laboratory indicators associated with intestinal involvement severity

**Figure 5.**
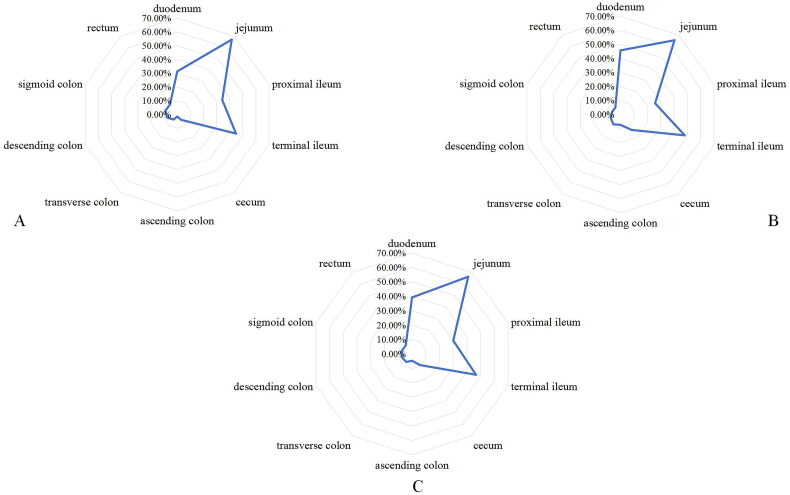
The distribution of affected intestinal segments. The three pictures represent children, adults and all age group, respectively.

For the assessment of involvement in affected intestinal segments, the evaluations by the two physicians achieved good and excellent agreement (Kappa value range from 0.699 to 1.000). The distribution of affected intestinal segments was shown in Figure 5. For intestinal complications, only a boy had ileo-ileal intussusception (Figure 1).

#### All age group

Patients of all age were grouped according to the aforementioned method (Table 2): mild involvement group (*n* = 76) and severe involvement group (*n* = 72). In the severe involvement group compared to the mild involvement group, D-dimer (7.97 mg/L vs. 4.37 mg/L, *p <* .001), WBC (15.45 × 10^9^/L vs. 13.11 × 10^9^/L, *p* = .021), and NLR (8.99 vs. 5.86, *p* = .006) were significantly elevated. While Alb was significantly decreased (35.10 g/L vs. 38.00 g/L, *p* = 0.002). After multiple imputation of missing values, a univariate logistic regression analysis was conducted. The results were shown in Table 3. Variables with a *p* < .1 were included in the multivariate logistic regression analysis. It was revealed that elevated D-dimer [odds ratio (OR)=1.104, *p* = .016] was an independent risk factor for severe intestinal involvement (Table 3).

**Table 2.** Laboratory indicators for different patients.

Item	Mild intestinal involvement	Severe intestinal involvement	*p*
All age group	*n* = 76	*n* = 72	
D-dimer (mg/L)	4.37 (2.18, 7.74) (n = 69)	7.97 (5.24, 11.04) (n = 62)	.000
CRP (mg/L)	32.40 (8.10, 68.10) (n = 63)	48.30 (21.80, 79.73) (n = 58)	.058
ESR (mm/h)	11.00(6.00, 14.75) (n = 68)	11.00 (6.00, 16.00) (n = 61)	.753
Alb (g/L)	38.00 (33.50, 40.60)(n = 75)	35.10 (29.85, 38.00) (n = 72)	.002
Hb (g/L)	136.07 ± 18.34	135.42 ± 26.42	.862
WBC (×10^9^/L)	13.11 (10.32, 17.69)	15.45 (11.25, 24.33)	.021
NLR	5.86 (4.04, 9.80)	8.99 (5.38, 15.36)	.006
MPV (fl)	10.22 ± 1.24	10.18 ± 1.26	.854
Children	*n* = 36	*n* = 31	
D-dimer (mg/L)	3.77 (1.56, 6.56) (*n* = 33)	7.29 (5.43, 11.66) (*n* = 25)	.001
CRP (mg/L)	27.70 (7.10, 69.10) (*n* = 27)	45.80 (17.00, 78.80) (*n* = 23)	.381
ESR (mm/h)	8 (6.00, 13.5) (*n* = 33)	8 (6, 12.75) (*n* = 24)	.782
Alb (g/L)	36.80 ± 6.23	34.01 ± 5.41	.058
Hb (g/L)	130.42 ± 16.96	130.1 ± 24.31	.950
WBC (×10^9^/L)	13.70 (11.70, 18.56)	17.06 (12.8, 28.09)	.018
NLR	5.86 (2.82, 9.49)	8.09 (4.97, 16.45)	.065
MPV(fl)	9.86 ± 1.35	9.82 ± 1.22	.894
Adults	*n* = 40	*n* = 41	
D-dimer (mg/L)	5.03 (2.58, 7.92) (*n* = 36)	8.07 (4.45, 10.84) (*n* = 37)	.011
CRP (mg/L)	35.60 (9.18, 66.25) (*n* = 36)	55.30 (26.10, 82.50) (*n* = 35)	.098
ESR (mm/h)	11.00 (7.00, 18.00) (*n* = 35)	13.00 (7.00, 19.00) (*n* = 37)	.676
Alb (g/L)	38.20 (33.80, 40.40) (*n* = 39)	35.80 (26.85, 38.15) (*n* = 41)	.014
Hb (g/L)	142.50 (123.00, 156.00)	140.00 (128.00, 156.00)	.940
WBC (×10^9^/L)	12.41 (9.92, 17.41)	14.06 (10.75, 20.33)	.247
NLR	6.06 (4.21, 11.37)	9.15 (6.22, 15.34)	.045
MPV (fl)	10.35 (9.80, 11.15)	10.10 (9.60, 11.20)	.505

CRP: C-reactive protein; ESR: erythrocyte sedimentation rate; Alb: albumin; Hb: hemoglobin; WBC: white blood cell; NLR: neutrophil-to-lymphocyte ratio; MPV: mean platelet volume.

**Table 3.** Results of logistics regression analysis for different patients.

Item	Univariate logistic regression analysis			Multivariate logistic regression analysis		
	OR	95%CI	*P*	OR	95%CI	*p*
All age group						
D-dimer (mg/L)	1.146	1.063–1.235	0.000	1.104	1.018–1.196	.016
CRP (mg/L)	1.006	0.998–1.014	0.124			
ESR (mm/h)	1.006	0.986–1.025	0.571			
Alb (g/L)	0.919	0.868–0.972	0.003	0.965	0.903–1.032	.296
Hb (g/L)	0.999	0.984–1.013	0.861			
WBC (×10^9^/L)	1.072	1.020–1.126	0.006	1.026	0.967–1.087	.399
NLR	1.075	1.017–1.136	0.011	1.039	0.972–1.111	.261
MPV (fl)	0.976	0.752–1.265	0.853			
Children						
D-dimer (mg/L)	1.204	1.058–1.370	0.005	1.181	1.013–1.377	.033
CRP (mg/L)	1.008	0.997–1.020	0.166			
ESR (mm/h)	1.002	0.979–1.025	0.887			
Alb (g/L)	0.92	0.842–1.005	0.064	1.011	0.906–1.129	.841
Hb (g/L)	0.999	0.976–1.023	0.949			
WBC (×10^9^/L)	1.094	1.016–1.178	0.018	1.048	0.963–1.141	.276
NLR	1.073	0.990–1.163	0.085	1.039	0.939–1.150	.460
MPV (fl)	0.974	0.667–1.422	0.892			
Adults						
D-dimer (mg/L)	1.093	1.002–1.093	0.045	1.056	0.974–1.145	.185
CRP (mg/L)	1.005	0.995–1.015	0.335			
ESR (mm/h)	1.009	0.973–1.046	0.64			
Alb (g/L)	0.912	0.845–0.983	0.016	0.084	0.857–1.010	.930
Hb (g/L)	0.997	0.978–1.016	0.740			
WBC (×10^9^/L)	1.059	0.987–1.137	0.111			
NLR	1.075	0.995–1.161	0.067	1.049	0.965–1.140	.262
MPV (fl)	0.935	0.636–1.375	0.733			

OR: odds ratio; CI: confidence interval; CRP: C-reactive protein; ESR: erythrocyte sedimentation rate; Alb: albumin; Hb: hemoglobin; WBC: white blood cell; NLR: neutrophil-to-lymphocyte ratio; MPV: mean platelet volume.

#### Children

Among the 67 patients in the children group, there were 36 patients in the mild involvement group and 31 patients in the severe involvement group. The specific results were shown in Table 2. In the severe involvement group, D-dimer (7.29 mg/L vs. 3.77 mg/L, *p* = .001) and WBC (17.06 × 10^9^/L vs. 13.70 × 10^9^ cells/L, *p* = .018) were significantly higher compared to the mild involvement group. The univariate logistic regression analysis showed that elevated D-dimer (OR = 1.204, *p* = .005) and WBC (OR = 1.094, *p* = .018) were associated with severe intestinal involvement (Table 3). The multivariable logistic regression analysis showed that elevated D-dimer (OR = 1.181, *p* = .033) was an independent risk factor for severe intestinal involvement (Table 3).

The results for the adult group can be found in supplementary material.

### Differences between different classifications based on the sites of intestinal involvement

According to the definition in the method above, among the 148 patients, the specific patient numbers of the four types were as follows: L1 type (*n* = 56; mild involvement:34, severe involvement:22), L2 type (*n* = 29; mild involvement:25, severe involvement:4), L3 type (*n* = 54; mild involvement:12, severe involvement:42), and L4 type (*n* = 9; mild involvement:5, severe involvement:4). Due to the small number of patients in the L4 type, this study only included patients of L1-L3 types in the analysis. The specific results were shown in Table 4. There were no significant statistical differences among the three groups in terms of age, gender, initial presentation of abdominal symptoms, gastrointestinal bleeding, abdominal pain, diarrhea, nausea and vomiting, skin purpura, and renal involvement. However, there was a significant difference in hospitalization duration among the three groups (L1 type: 12.00 d, L2 type: 11.00 d, L3 type: 16.50 d, *p* = .010). Pairwise comparisons revealed that the hospitalization duration was significantly increased in the L3 type compared to the L1 type (adjusted *p* = .021). While no significant statistical differences were found between the L1 and L2 types or between the L2 and L3 types (adjusted *p* > .99; adjusted *p* = .052). However, when grouped by age, there were no statistical differences among all factors (Supplementary Table 1, Supple­mentary Table 2).

**Table 4.** Differences among different subtypes of IgAV patients with intestinal involvement for all age group.

Item	L1 (*n* = 56)	L2 (*n* = 29)	L3 (*n* = 54)	*p*
Age (year)	20.00 (9.25, 36.25)	19.00 (14.00, 40.50)	18.00 (13.00, 35.25)	.932
Gender (male)	39 (69.6%)	22 (75.9%)	41 (75.9%)	0.715
Gastrointestinal bleeding	38 (67.9%)	23 (79.3%)	46 (85.2%)	.092
Abdominal pain	55 (98.2%)	28 (96.6%)	51 (94.4%)	.569
Diarrhea	39 (69.6%)	24 (82.8%)	43 (79.6%)	.306
Nausea and vomiting	24 (42.9%)	13 (44.8%)	30 (55.6%)	.379
Skin purpura	47 (83.9%)	26 (89.7%)	45 (83.3%)	.738
Abdominal symptoms as the initial presentation	18 (32.1%)	9 (31.0%)	23 (42.6%)	.429
Renal involvement	22 (39.3%)	12 (41.4%)	30 (55.6%)	.197
Length of hospital stay (day)	12.00 (8.00, 17.75)	11.00 (8.00, 17.50)	16.50 (11.00, 23.25)	.010

IgAV: immunoglobulin A vasculitis.

## Discussion

Joint pain and renal involvement were the primary clinical differences between children and adults, with no significant variations in other symptoms. The consistency of CT-based assessment of intestinal involvement provided additional and objective information for identifying populations that require special attention in clinical diagnosis and treatment. Elevated D-dimer level was an independent risk factor for severe intestinal involvement in IgAV patients. L3 type patients (involvement of the duodenum and/or jejunum and ileum) had longer hospital stays and should be closely monitored.

Literature showed the incidence rate of IgAV in children was 2 to 33 times higher than that in adults [18]. The occurrence rate of intestinal involvement does not show significant differences [19]. But the present study found a higher proportion of adults compared to children (67 vs.81). This may be related to the fact that the pediatric center at Tongji Hospital was established in 2022 and primarily handles critically ill children. So, most children with IgAV would choose specialized pediatric hospitals. The results of this study showed that although there was no significant difference in the occurrence rate of gastrointestinal bleeding between children and adult, the proportion of adult patients experiencing melena was higher (48.1% vs. 72.6%). This suggested that adults had a greater amount of gastrointestinal bleeding. A previous study reported a higher rate of renal involvement in adults compared to children [20], which was consistent with the results of the present study. This suggested that adult patients should pay more attention to the detection of renal involvement.

Research on renal involvement in IgAV is comprehensive and receives significant attention, whereas gastrointestinal involvement is often overlooked. This discrepancy may account for the lower incidence of intussusception observed in this study (1/148) compared to the reported literature (0.7% to 13.6% [5]). The gastrointestinal manifestations in IgAV patients can be highly covert and subjective, varying greatly depending on individual tolerance. Therefore, there is a strong subjectivity in the assessment. However, it is crucial to identify severe intestinal involvement. It indicates the need for a change in treatment approach. A guideline recommends that for patients with severe intestinal involvement, the administration of prednisolone should be switched from oral to intravenous, with an increased dosage [12].

Identifying laboratory biomarkers that correlate with intestinal severity in IgAV allows clinicians to precisely assess disease severity and customize treatment plans, enhancing patient outcomes. Unlike previous studies that defined severe intestinal involvement, such as severe colicky abdominal pain, gastrointestinal bleeding and complications like intussusception [9–11], this study defined the severity of intestinal involvement based on the extent of intestinal involvement shown in the initial CT scan at the time of hospital admission. This can provide a more objective reflection of the disease’s status. This study found that among the 148 included patients, the group with severe intestinal involvement had higher levels of D-dimer, WBC, NLR, and lower albumin level. This indicated more pronounced hypercoagulability, inflammatory response, and poorer nutritional status in the severe intestinal involvement group. Multivariate logistic regression analysis identified D-dimer as an independent risk factor for predicting severe intestinal involvement. This was related to a more extensive involvement of the intestine in the severe intestinal involvement group. After grouping by age, D-dimer also was an independent risk factor for predicting severe intestinal involvement for children. Elevated D-dimer levels have been confirmed by researchers as an indicator of disease severity. A study by Mario Sestan et al. showed that D-dimer levels were higher in patients with severe skin involvement [21]. A similar conclusion was drawn in a study by Minhui Li et al. investigating renal involvement in IgAV patients [22]. These findings underscored the potential utility of D-dimer as a biomarker for disease severity, which could guide risk stratification and individualized treatment plans in clinical practice. Future research could focus on establishing standardized D-dimer thresholds for predicting complications in IgAV and determining its role in monitoring disease progression.

In addition to grouping based on the extent of intestinal involvement, this study also grouped patients based on the site of involvement. Previous studies on intestinal diseases found that different lesion locations lead to different distributions of disease populations, clinical manifestations, and prognoses [23]. For Crohn’s disease (CD), the disease locations and behaviors defined by the Montreal classification were closely associated with clinical symptoms and the natural course of the disease. Referring to the Montreal classification, this study classified IgAV patients with intestinal involvement into four types. Only 9 (6.1%) patients were found to be involved limited to the colorectum, suggesting that the majority of patients have small intestine involvement. And it predominantly located in the jejunum. This was different from other inflammatory intestine diseases. For example, CD and intestinal Behçet’s disease were most involved in the ileocecal region [24]. What is more, the present study indicated that 83 out of 126 patients (65.9%) exhibited petaloid bowel wall thickening, and 58 out of 148 patients (39.2%) exhibited target sign. Therefore, CT-based intestinal evaluation can aid in the diagnosis of the disease, especially for patients presenting with predominantly abdominal symptoms and without typical purpuric skin rash. The first three types of patients showed similar age and gender distributions. And there were no significant differences in the abdominal symptoms, skin involvement, and renal involvement. This indicated that the distribution of patient populations and clinical symptoms did not significantly differ among different types of patients. However, patients with the L3 type had longer hospital stays, suggesting a more severe overall disease condition that requires a longer duration of individual treatment cycles. This was consistent with clinical experience, as L3 type patients have a extensive range of intestinal involvement, naturally resulting in a longer recovery time. While this was related to the efficacy of diagnosis and treatment, it was likely that more extensive intestinal involvement indicated a more severe disease state. However, the relationship and causality between the extent of intestinal involvement and known indicators of severity, such as widespread rash and renal involvement, remain inconclusive based on this study. This warrants further investigation by researchers.

There were some limitations to this study. First, it was a retrospective study. We will consider conducting a prospective study in the future to strengthen the conclusions drawn from the data. Secondly, this study did not compare the differences in race and geographic distribution of patients to assess the generalizability of the study results. This was related to the fact that the patients in this study were primarily Han Chinese located in the central region of the country. In subsequent studies, conducted either by our team or other research groups, we plan to leverage multicenter data or focus on varying ethnic populations to thoroughly evaluate the generalizability of the results. Thirdly, some patients had incomplete laboratory data. Due to the small number of cases, excluding patients with missing data would significantly reduce the number of cases and result in the loss of some useful information. Therefore, multiple imputation was used to fill in missing values for data analysis. Using multiple imputation to address missing data is a common method among statisticians, as evidenced by its application in the studies conducted by Carson et al. and Hoge et al. [25,26]. What is more, this study did not conduct a comparison of disease prognosis due to the self-limiting nature of intestinal involvement of IgAV and the difficulty in obtaining follow-up data. This resulted in limited information on disease recurrence rates.

## Conclusion

This study demonstrated that, except for joint pain and renal involvement, there were no significant differences in clinical symptoms between children and adults. CT provided more objective information for intestinal evaluation. So, it is crucial to prioritize the evaluation of patients’ imaging manifestations in the diagnosis and treatment of IgAV. Additionally, elevated D-dimer level was associated with severe intestinal involvement. It is important for clinicians to pay attention to the D-dimer levels of patients. Patients with involvement of the entire small intestine demonstrated a more severe disease condition. Clinicians should be particularly attentive to the prognosis and outcomes of this patient group.

## Supplementary Material

Supplemental Material

## Data Availability

The data will be shared upon reasonable request made to the corresponding author or any particular author of the manuscript.
