# Supplementary material for: The extent of intestinal involvement is closely related to the severity of IgAV: a risk stratification study based on CT
Source: Ann Med. 2025 Feb 7;57(1):2462260. doi: 10.1080/07853890.2025.2462260 (PMC11809178; doi:10.1080/07853890.2025.2462260)
Supplement: Supplemental Material [file IANN_A_2462260_SM2931.zip › Suppl_Data/Supplementary material (38).docx]

The adult group consisted of 81 patients: 40 patients in the mild involvement group and 41 patients in the severe involvement group. The specific results are shown in Table 2. The results showed that in the severe involvement subgroup, D-dimer [8.07 (4.45, 10.84) mg/L (n=37) vs. 5.03 (2.58, 7.92) mg/L (n=36)] and NLR [9.15 (6.22, 15.34) vs. 6.06 (4.21, 11.37), *P*=0.045] were significantly elevated. While Alb [35.80 (26.85, 38.15) g/L (n=41) vs. 38.20 (33.80, 40.40) g/L (n=39), *P*=0.014] was significantly reduced. The univariate logistic regression analysis showed that elevated D-dimer (OR=1.093, 95%CI: 1.002-1.093, *P*=0.045) and decreased Alb (OR=0.912, 95%CI: 0.845-0.983, *P*=0.016) were associated with severe intestinal involvement (Table 3). However, the multivariable logistic regression analysis indicated that none of the indicators were independent risk factors for severe intestinal involvement (Table 3).
