# Supplementary material for: The extent of intestinal involvement is closely related to the severity of IgAV: a risk stratification study based on CT
Source: Ann Med. 2025 Feb 7;57(1):2462260. doi: 10.1080/07853890.2025.2462260 (PMC11809178; doi:10.1080/07853890.2025.2462260)
Supplement: Supplemental Material [file IANN_A_2462260_SM2931.zip › Suppl_Data/Supplementary Table 1 (11).docx]

Supplementary Table 1. Differences among different subtypes of IgAV patients with intestinal involvement for children.

| Item | L1 (n=23) | L2 (n=14) | L3 (n=26) | *P* |
| --- | --- | --- | --- | --- |
| Age (year) | 8.00 (5.00, 14.00) | 14.00 (8.50, 15.00) | 13.00 (6.75, 15.25) | 0.086 |
| Gender (male) | 15 (65.2%) | 10 (71.4) | 19 (73.1%) | 0.827 |
| Gastrointestinal bleeding | 17 (73.9%) | 11 (78.6%) | 22 (84.6%) | 0.650 |
| Abdominal pain | 22 (95.7%) | 13 (92.9%) | 24 (92.3%) | 0.883 |
| Diarrhea | 14 (60.9%) | 9 (64.3%) | 22 (84.6%) | 0.148 |
| Nausea and vomiting | 11 (47.8%) | 7 (50.0%) | 14 (53.8%) | 0.913 |
| Skin purpura | 19 (82.6%) | 13 (92.9%) | 20 (76.9%) | 0.449 |
| Abdominal symptoms as the initial presentation | 8 (34.8%) | 5 (35.7%) | 12 (46.2%) | 0.778 |
| Renal involvement | 5 (21.7%) | 6 (42.9%) | 12 (46.2%) | 0.178 |
| Length of hospital stay (day) | 12.00 (8.00, 17.00) | 12.50 (8.00, 22.75) | 18.00 (10.75, 22.50) | 0.103 |

IgAV, imunoglobulin A vasculitis.
