# Supplementary material for: The extent of intestinal involvement is closely related to the severity of IgAV: a risk stratification study based on CT
Source: Ann Med. 2025 Feb 7;57(1):2462260. doi: 10.1080/07853890.2025.2462260 (PMC11809178; doi:10.1080/07853890.2025.2462260)
Supplement: Supplemental Material [file IANN_A_2462260_SM2931.zip › Suppl_Data/Supplementary Table 2 (2).docx]

Supplementary Table 2. Differences among different subtypes of IgAV patients with intestinal involvement for adults.

| Item | L1 (n=33) | L2 (n=15) | L3 (n=28) | *P* |
| --- | --- | --- | --- | --- |
| Age (year) | 32.00 (23.50, 54.50) | 40.00 (25.00, 55.00) | 32.50 (21.25, 48.50) | 0.717 |
| Gender (male) | 24 (72.7%) | 12 (80.0%) | 22(78.6%) | 0.808 |
| Gastrointestinal bleeding | 21 (63.6%) | 12 (80.0%) | 24(85.7%) | 0.123 |
| Abdominal pain | 33 (100%) | 15 (100%) | 27(96.4%) | 0.420 |
| Diarrhea | 25 (75.8%) | 15 (100%) | 21(75.0%) | 0.100 |
| Nausea and vomiting | 13 (39.4%) | 6 (40.0%) | 16(57.1%) | 0.333 |
| Skin purpura | 28 (84.8%) | 13 (86.7%) | 25(89.3%) | 0.877 |
| Abdominal symptoms as the initial presentation | 10 (30.3%) | 4 (26.7%) | 11(39.3%) | 0.643 |
| Renal involvement | 17 (51.5%) | 6 (40.0%) | 18(64.3%) | 0.293 |
| Length of hospital stay (day) | 12.00 (8.00, 18.00) | 11.00 (8.00, 14.00) | 14.50 (11.00, 23.75) | 0.094 |

IgAV, imunoglobulin A vasculitis.
